# Histone 2B Facilitates Plasminogen-Enhanced Endothelial Migration through Protease-Activated Receptor 1 (PAR1) and Protease-Activated Receptor 2 (PAR2)

**DOI:** 10.3390/biom12020211

**Published:** 2022-01-26

**Authors:** Mitali Das, Sujay Subbayya Ithychanda, Edward F. Plow

**Affiliations:** Department of Cardiovascular and Metabolic Sciences, Lerner Research Institute, Cleveland Clinic, Cleveland, OH 44195, USA; ithychs@ccf.org (S.S.I.); plowe@ccf.org (E.F.P.)

**Keywords:** endothelial cells, histone 2B, migration, plasminogen, protease-activated receptors

## Abstract

Plasminogen and its multiple receptors have been implicated in the responses of many different cell types. Among these receptors, histone 2B (H2B) has been shown to play a prominent role in macrophage responses. The contribution of H2B to plasminogen-induced endothelial migration, an event relevant to wound healing and angiogenesis, is unknown. Plasminogen enhanced the migration of endothelial cells, which was inhibited by both Protease-Activated Receptor-1 (PAR1) and 2 (PAR2) antagonists. H2B was detected on viable endothelial cells of venous and arterial origin, and an antibody to H2B that blocks plasminogen binding also inhibited the plasminogen-dependent migration by these cells. The antibody blockade was as effective as PAR1 or PAR2 antagonists in inhibiting endothelial cell migration. In pull-down experiments, H2B formed a complex with both PAR1 and PAR2 but not β3 integrin, another receptor implicated in endothelial migration in the presence of plasminogen. H2B was found to be associated with clathrin adapator protein, AP2µ (clathrin AP2µ) and β-arrestin2, which are central to the internationalization/signaling machinery of the PARs. These associations with PAR1-clathrin adaptor AP2µ- and PAR2-β-arrestin2-dependent internalization/signaling pathways provide a mechanism to link plasminogen to responses such as wound healing and angiogenesis.

## 1. Introduction

Multiple cell surface molecules serve as plasminogen receptors (PlgRs), and the surfaces of many cells, including monocytoid cells, endothelial cells and tumor cells, are decorated with multiple PlgRs [1,2,3,4,5,6,7]. Many of these PlgR bear C-terminal lysines that interact with the lysine binding sites within the kringle domains of Plg. By tethering Plg and plasmin to PlgRs, cells are able to harness the proteolytic activity of plasmin (Plm) to facilitate processes such as migration and invasion. Several of the identified PlgRs lack transmembrane domains and yet are able to induce intracellular signaling responses, such as cytokine production and gene expression [8,9,10,11]. In this category of PlgRs, endothelial cell responses have been attributed to Annexin 2 [12,13,14], p11 [15,16,17] and Enolase-1 (ENO-1) [6,18].

Histone 2B (H2B) is a major PlgR on leukocytes [19,20]. Its expression level is markedly upregulated when monocytoid cells are stimulated with cellular activators [19] and H2B has been shown to influence several Plg-dependent responses, including inflammatory cell recruitment [10,21], the phagocytosis of microorganisms and damaged cells [11], and the uptake of oxidized phospholipids [10]. Concomitant regulation of gene expression in association with these responses has been demonstrated both in vitro and in vivo [10,11].

Protease-activated receptors (PARs) are G protein-coupled receptors (GPCRs) that are expressed on diverse cell types and initiate many different cell functions including migration and invasion [22,23,24,25]. PARs (PAR1, -2, -3 and -4) are activated by a unique mechanism: distinct proteases cleave at specific sites in the extracellular N-terminal domain of PARs and the new amino (NH2)-terminal sequence acts as a tethered ligand to induce intracellular signaling [26,27,28]. The ensuing signaling is terminated when GPCRs are rapidly desensitized followed by binding to β-arrestins. Cytosolic β-arrestins are recruited to the plasma membrane where they target phosphorylated GPCRs to the clathrin-mediated endocytic machinery of the cell. PAR1 binds to the clathrin adaptor protein, AP2, whereas PAR2 connects to the endocytic machinery via β-arrestin 2 [29,30].

PAR1 and PAR2 were both involved in the migration and metastasis of tumor cells when stimulated with thrombin [24]. Activation of PAR2 was shown to induce angiogenesis in a murine model of hind limb ischemia [31] while hypoxia-induced angiogenesis was driven by PAR2 and not PAR1 in a mouse model [32]. Although PAR1 were shown to be involved in Plm-mediated mononuclear recruitment in murine models [33], migration of human monocytes was via Annexin 2 and not PAR1 dependent [34]. Little is known regarding whether the transmembrane-lacking PlgRs engage PARs to influence endothelial function such as cell migration.

In a prior review, we reported that H2B is also present on the surface of endothelial cells [35]. However, its role in Plg-dependent endothelial cell migration, a response critical to angiogenesis, wound healing and tumor growth and metastasis, is entirely unknown. Although it is known that Plm promotes cell migration [23,36] and invasion [37,38], there is limited evidence linking it to PARs [22,32,39] and transmembrane-lacking PlgRs such as H2B in the migration of endothelial cells. We hypothesized that H2B could influence endothelial cell migration via the PAR1 and/or PAR2 pathway as both PAR1 and PAR2 are important in cell migration and invasion [24] and are abundantly expressed in endothelial cells [40]. Therefore, in this study, we sought to address the following specific questions: (i) What is the effect of Plg on the migration of endothelial cells? (ii) How is Plg-induced endothelial migration linked to the PARs? (iii) What is the role of H2B in the Plg-driven migration of endothelial cells? (iv) How does H2B-PAR-mediated signaling intersect in cell migration?

## 2. Materials and Methods

### 2.1. Cell Culture

Human umbilical vein endothelial cells (HUVECs) from single donors were purchased and cultured in endothelial cell growth medium (Cell Applications, San Diego, CA, USA). Early passage (1–3), single donor HUVECs were used for all experiments. Pulmonary Arterial Endothelial Cells (PAEC; passage 6 for Donor 1 and passage 7 for Donor 2) from donors without history of pulmonary hypertension were obtained from the Cleveland Clinic’s Biorepository [41]. PAEC were grown in MCDB105 medium (Sigma, Burlington, MA, USA) supplemented with 90 µg/mL heparin (Sigma, Burlington, MA), 90 µg/mL endothelial growth supplement (BD Biosciences, San Jose, CA, USA), 15 µg/mL glycine, 15 µg/mL KCl, 15% heat inactivated fetal bovine serum and normocin (Invitrogen, San Diego, CA, USA) as an antibacterial-antimycotic-antimycoplasmic agent.

### 2.2. Reagents and Antibodies

Human Glu-Plg was from Haematologic Technologies Inc (Essex Junction, VT, USA), PAR1 antagonist, RWJ56110, and PAR2 antagonist, Phenylalanine-Serine-Leucine-Leucine-Arginine-Tyrosine-NH2 (FSLLRY-NH2), were from Tocris Bioscience (Bristol, UK), irreversible Plm inhibitor, DVFL (D-Val-Phe-Lys chloromethylketone dihydrochloride) was from Millipore Sigma (Burlington, MA, USA). Peptides were dissolved in solvents/buffers according to the manufacturers’ instructions. Working stocks of these reagents were all diluted in (Dubelco’s Modified Eagle Medium (DMEM): F12 + 0.1% bovine serum albumin (BSA). The same medium was used to resuspend cells for experiments. Calpain inhibitor XI and phosphatase inhibitor cocktail set II were from Millipore Sigma (Burlington, MA, USA). Mouse monoclonal antibody (mAb) was raised against the C-terminal H2B peptide Ac-VTKYTSSK-COOH in the Cleveland Clinic hybridoma core. An ImmunoPure Fab preparation kit (ThermoFisher Scientific, Waltham, MA, USA) was used to prepare Fab fragments of this mAb, and purity of the Fab fragments was determined using sodium dodecylsulfate polyacrylamide gel electrophoresis (SDS-PAGE). Rabbit antibodies against H2B, Annexin 2, p11 and Enolase-1 (ENO-1) have been previously described [20,42]. The following mAb were used at manufacturer-recommended dilutions: (a) Immunoblotting-AP2μ (BD Biosciences, San Jose, CA, USA), PAR1 (R&D Systems, Minneapolis, MN, USA), PAR2 (SAM11 clone (Millipore Sigma, Burlington, MA), β3 integrin (BD Biosciences, San Jose, CA, USA), β-arrestin 1 (BD transduction Laboratories, San Jose, CA, USA), β-arrestin 2 (Abcam, Cambridge, UK) and horse-radish peroxidase-conjugated anti-mouse or anti-rabbit secondary antibodies (Millipore Sigma, Burlington, MA, USA). (b) Anti-mouse Fab fragment (Rockland Inc, Limerick, PA, USA), rabbit polyclonal PAR1 (immunoprecipitation; Abnova, Taipei, Taiwan), mAb to PAR1 (WEDE15 clone, flow cytometry; Beckman Coulter, Brea, CA, USA), to PAR2 (SAM11 clone, flow cytometry; Millipore Sigma, Burlington, MA, USA), rabbit monoclonal PAR2 (immunoprecipitation; Cell Signaling Technology, Danvers, MA, USA). Ultraviolet (UV)-excitable LIVE DEAD dye, anti-mouse or anti-rabbit red fluorescent phycoerythrin (R-PE), Alexa Fluor 488 secondary antibodies (IgG Fab) were from ThermoFisher Scientific (Waltham, MA, USA).

### 2.3. Endothelial Cell Migration Assays

For all cell types and in all assays, cells were washed with phosphate buffered saline (PBS) and then dissociated with non-enzymatic Cell Dissociation Buffer (ThermoFisher Scientific, Waltham, MA, USA). In these assays, DMEM: F12 and 0.1% BSA was used to resuspend cells and as a diluent for reagent additions. Cells were washed 2 times with PBS and starved overnight in DMEM: F12 + 1% fetal bovine serum (FBS) at 37 °C in a tissue culture incubator. After washing with PBS, equal amounts of HUVECs (non-aggregated, single cell suspensions) were left untreated, 200 nM human Glu-Plg was added, either alone or with 20 nM Plm inhibitor, to 10 µM PAR1 or 10 µM PAR2 antagonist for 10 min at 37 °C. For antibody-blocking experiments, cells were incubated with 20 µg/mL mouse Fab or mouse anti-H2B Fab for 30 min at 37 °C and left untreated or Plg added. Antibody-blocked cells were also incubated with Plg and PAR antagonists. Using 8 μm pore transwell inserts and 1% FBS as a chemoattractant, 18–20 hour migration assays were processed using CyQuant Cell Proliferation Assay kits ThermoFisher Scientific, Waltham, MA, USA) as described [20]. Migrated cells were quantified based on fluorescence from the signal of CyQuant reagent after subtracting background fluorescence from control wells. For each condition, at least triplicates were used.

### 2.4. Flow Cytometry

Dissociated cells were treated with a UV-excitable live–dead dye to exclude dead cells from analyses. Cells were incubated with diluted primary antibodies on ice for 30 min, washed and then incubated with Alexa-Fluor 488- or R-PE-labeled anti-mouse or anti-rabbit IgG on ice for 30 min. Cells were fixed with 2% paraformaldehyde, washed and resuspended in PBS and analyzed on a LSRII (BD Biosciences, San Jose, CA, USA). Flow cytometric data were analyzed as previously described [42,43] using the FlowJo software version IX (TreeStar Inc, Ashland, OR, USA). In all experiments using Plg, cells were processed as described [43]. Surface expression of proteins was calculated by subtracting the fluorescence from secondary antibody alone and expressed as Relative Fluorescence Intensity (RFI). Antibodies against H2B, p11, ENO-1, and Annexin 2 were previously described [20].

### 2.5. Immunoblotting

Cells dissociated with Cell Dissociation Buffer were centrifuged at 13,000× g for 5 min, and cell pellets were lysed in buffer containing 50 mM Tris-HCl (pH 7.4), 150 mM NaCl, 1% Triton X-100, protease inhibitor tablet (Roche Applied Science, Penzberg, Germany), phenylmethylsulfonyl fluoride, calpain inhibitor XI and phosphatase inhibitor cocktail set II (Millipore Sigma, Burlington, MA, USA) on ice for 30 min. After centrifugation for 30 min at 13,000× g at 4 °C, supernatants were used for immunoprecipitation, pulldown or immunoblotting assays. For immunoblotting, lysates or precipitates from glutathione-S-transferase (GST)-pulldown or immunoprecipitation assays were resuspended in Laemmli buffer (Millipore Sigma, Burlington, MA, USA) and boiled for 5 min at 95 °C. Proteins was resolved by SDS-PAGE, transferred onto polyvinylidene difluoride (PVDF) membranes (Bio-Rad, Hercules, CA, USA), followed by blocking in 5% BSA in PBS-Tween 20 (PBS-T) for 1 h at ambient temperature. Overnight incubation with the antibody of interest was followed by washing the membranes three times with PBS-T. Subsequent incubation with appropriate horse radish peroxidase antibody for 1 h at room temperature was followed by washing with PBS-T for three times. Membranes were subjected to autoradiography to detect proteins.

### 2.6. GST Pulldown Assays

Starved HUVEC cells were either untreated or treated with 200 nM human Glu-Plg as for migration assays and lysed using the same methodology as for immunoblotting. GST pulldown assays were performed using equal amounts of bacterially expressed GST-H2B or GST alone (control). Lysates used for GST pulldown assays were supplemented with 1 mM dithiothreitol (DTT) to prevent dimerization of GST proteins. Similar amount of total protein in each lysate were incubated with equal amounts of GST or GST fusion proteins and equal volume of glutathione-Sepharose 4B slurry for 18 h at 4 °C. Precipitates were collected after centrifugation (13000× g for 5 min at 4 °C), washing and boiling in Laemmli buffer. Interactions between GST alone or GST fusion proteins and the eluates were analyzed via immunoblotting under reducing conditions. PAR1, PAR2, AP2µ, β3 integrin, β-arrestin 2 and β-arrestin 1 and H2B were probed with the antibodies described above. Similar loading of GST proteins were monitored by Coomassie Blue or GelCode staining of gels.

### 2.7. Immunoprecipitation

Cell lysates containing equal amount of total protein were precleared with washed Protein A/G PLUS beads (Santa Cruz Biotechnology, Dallas, Texas, TX, USA) for 45 min at 4 °C. Supernatants were incubated with equal amount of control IgG or antibody to protein of interest at 4 °C for 4 h. This mixture was then allowed to bind to a new set of washed Protein A/G PLUS beads, overnight at 4 °C. The beads were pelleted by centrifugation at 13000× g for 5 min at 4 °C and washed thrice with PBS. Finally, the beads were resuspended in Laemmli buffer and processed for immunoblotting. AP2µ, and ENO-1 were probed with the antibodies described above.

### 2.8. Statistical Methods

Analysis of Variance (ANOVA) was performed for groups with multiple comparisons with Holm–Sidak or Tukey tests. For comparisons between two groups, a paired t-test was employed. In all analyses, a *p* < 0.05 was used to indicate significance.

## 3. Results

### 3.1. Plg Supports Migration in Endothelial Cells

**Figure 1:**
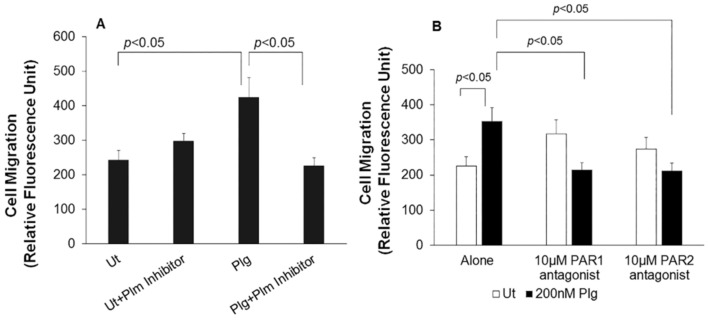
Plasminogen enhances endothelial migration using PAR1 and PAR2. Starved HUVECs, untreated (Ut) or treated with Plg, were allowed to migrate as described in “Material and Methods”. “Alone” denotes HUVECs in diluent of DMEM: F12 + 0.1% BSA. Cells, the Plm inhibitor and PAR antagonists were resuspended in this same medium. (**A**) Plg induced significantly higher endothelial cell migration compared to Ut HUVECs, and this increase was significantly inhibited by an irreversible Plm inhibitor, D-Val-Phe-Lys chloromethylketone dihydrochloride. (**B**) Effects of PAR1 and PAR2 antagonists on HUVEC migration in the presence or absence of Plg. Data are representative of 3 independent experiments, each containing at least triplicates. Values are means ± S.E.

Prior studies have established that PlgRs in general [5,7,44] and H2B in particular play roles in inflammatory cell migration [20,45]; however, its role in endothelial cell migration is totally unknown. Therefore, as a first step, HUVECs were treated with human Glu-Plg (200 nM) and allowed to migrate towards 1% FBS in the lower chamber through 8 µm pores of Boyden transwell chambers. Plg enhanced endothelial cell migration by 75% (*p* < 0.05) compared to untreated (Ut) cells. An irreversible peptide inhibitor of Plm, DVFL (D-Val-Phe-Lys chloromethylketone dihydrochloride), suppressed migration to the level observed with untreated cells, demonstrating that conversion of Plg to Plm was necessary to enhance Plg-dependent HUVEC migration (Figure 1A).

Since Plm is capable of activating PARs [46], we tested whether PAR1 and PAR2 antagonists could blunt Plg-induced HUVEC migration. Indeed, both antagonists reduced migration by ~40% (Figure 1B), a statistically significant inhibition (*p* < 0.05). Unlike monocyte migration, which was dependent only on PAR1 [33], PAR1 and PAR2 supported Plg-dependent endothelial cell migration (Figure 1B). Of note, each PAR antagonist effectively suppressed migration to the level seen in the absence of added Plg, indicating overlapping roles of the two PARs in the migratory response.

### 3.2. Role of H2B in Plg-Induced HUVEC Migration

**Figure 2:**
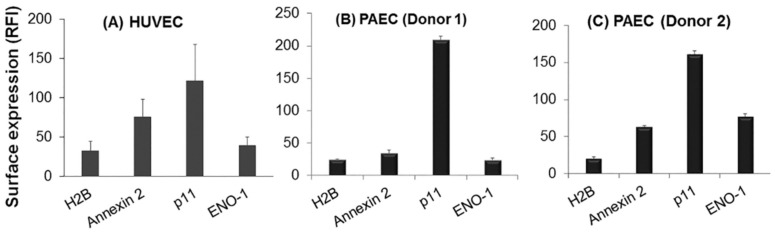
Surface expression of PlgRs in endothelial cells as detected by flow cytometry. H2B, as well as other well-established PlgRs, Annexin 2, p11 and ENO-1, are present on the surface of endothelial cells of venous (**A**) and arterial origin (**B**,**C**). Data are representative of HUVEC from multiple single donors and single PAEC experiments with at least 3 replicates in each analysis. Values are means ± S.E. mAb to H2B and rabbit anti-Annexin-2, p11 and ENO-1 were detected by flow cytometry on single cell suspensions of viable endothelial cells.

To consider the role of the H2B in Plg-dependent endothelial cell migration, we sought to first verify that this PlgR is present on the surface of viable endothelial cells. Viability dyes were used in flow cytometry to distinguish live from dead/necrotic cells since Plg has been linked to apoptosis [47]. Analyses were carried out on a population of cells that were viable, single and non-aggregated. H2B was detected, albeit at low and similar levels, on the surface of viable endothelial cells of both venous (HUVEC) and arterial (PAEC) origin (Figure 2). Compared to other PlgR lacking transmembrane domains, p11 was the most abundant on the surface of these endothelial cells, whereas Annexin 2 and ENO-1 were detected at lower levels, similar to that of H2B.

**Figure 3:**
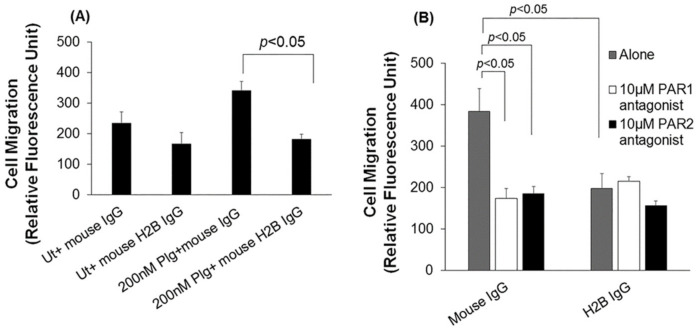
Plasminogen-induced endothelial cell migration depends on H2B, PAR1 and PAR2. Starved HUVECs, untreated (Ut) or treated with Plg, PAR1 or PAR2 antagonists, mouse Fab or Fab of mAb to H2B, either alone or in combinations, were allowed to migrate in a Boyden chamber. “Alone” denotes HUVECs with mouse IgG or anti-H2B alone. Mouse IgG, H2B IgG, PAR antagonists and cells were resuspended in DMEM: F12 + 0.1% BSA. (**A**) H2B blockade with Fab of anti-H2B significantly inhibited the migration of Plg-treated cells. (**B**) Effect of anti-H2B, PAR1 and PAR2 antagonists on migration facilitated by Plg. Data are representative of 3 independent experiments containing at least triplicates in each. Values are means ± S.E.

To determine if H2B contributed to Plg-dependent cell migration, HUVEC were either incubated with Fab fragments of mAb to H2B against the C-terminus of the H2B, an Fab fragment that was previously shown to specifically block Plg binding to H2B but not several other PlgR [20], or with a control Fab of anti-mouse IgG, and allowed to migrate in Boyden chamber assays. Control cells were left untreated or treated with Plg only. In these cells, Plg enhanced the migratory response by 45% compared to untreated cells in the presence of control mouse IgG Fab (Figure 3A). Anti-H2B Fab decreased the migration in Plg-treated cells by 47% (*p* < 0.05) and in untreated cells by 28% (Figure 3A).

We next sought to determine how H2B interfaces with the PARs in Plg-dependent cell migration. After preincubation with control mouse IgG or anti-H2B IgG, cells were treated with Plg, either alone or in combination with the PAR1 or PAR2 antagonists. Blockade of H2B with anti-H2B resulted in a ~50% decrease in Plg-mediated migration compared to Plg-treated cells subjected to anti-mouse IgG alone (*p* < 0.05). Both PAR1 and PAR2 antagonists inhibited Plg-induced HUVEC migration by greater than 50% (*p* < 0.05). When cells were pretreated with anti-H2B antibody, the PAR1 or PAR2 antagonists did not show further inhibition than that induced by the PAR1 and PAR2 antagonists alone. The PAR2 antagonist was slightly but not significantly more inhibitory than the anti-H2B alone. However, the PAR2 antagonist modestly but significantly (*p* < 0.05) diminished the response compared to that mediated by the PAR1 antagonist in the presence of the ant-H2B antibody. This pattern suggests that blockade of PAR1 and PAR2 inhibits events that overlap with the contribution of H2B to the Plg-stimulated migratory response (Figure 3B).

### 3.3. Interaction of H2B with PAR1 and PAR2 on HUVEC

**Figure 4:**
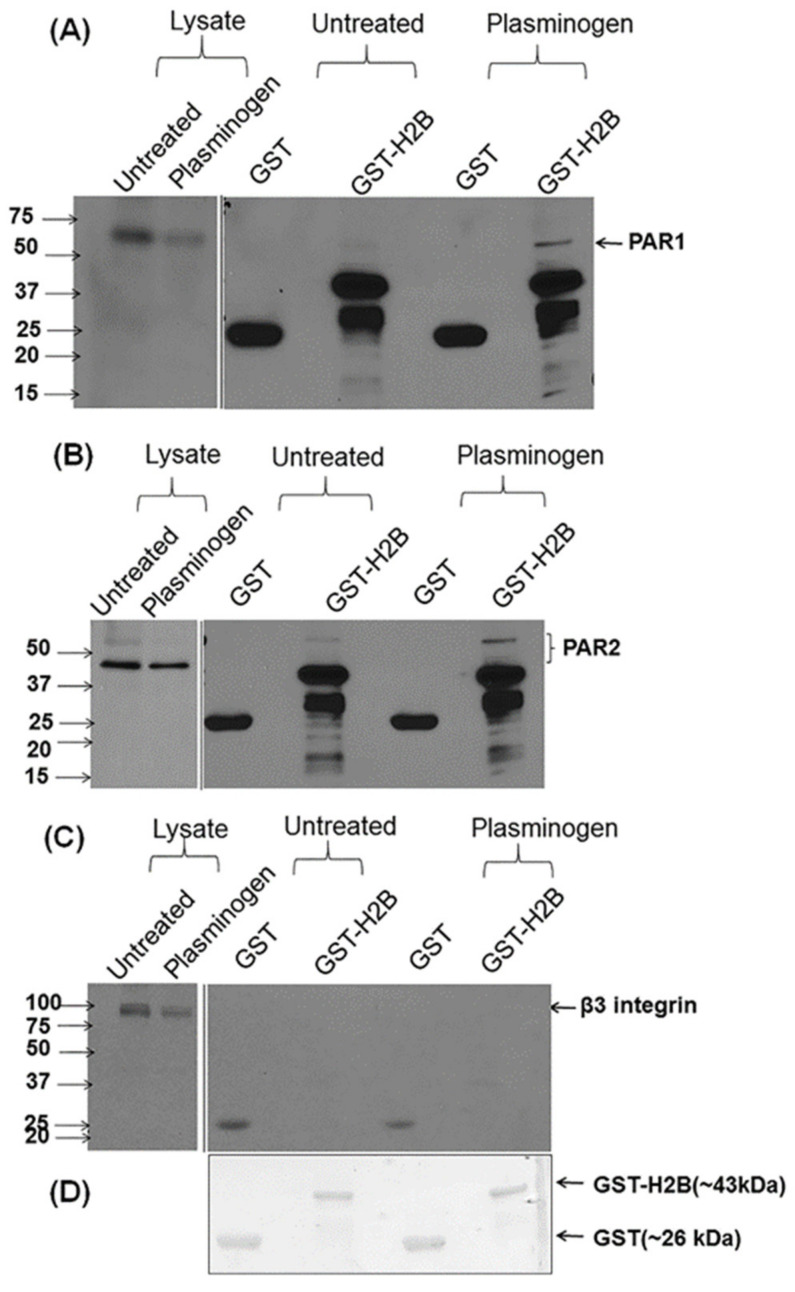
H2B associates with PARs and not with αvβ3, a transmembrane PlgR. GST-H2B pull-downs were performed on lysates of untreated (Ut) or Plg-stimulated HUVECs and probed by Western blots using mAbs to: (**A**) PAR1, (**B**) PAR2 and (**C**) the integrin β3 subunit. GST alone was used as a loading control. (**D**) Equal loading of GST and GST-H2B is shown on Coomassie stained gels. Solid lines between gels denote the position of the molecular weight ladder, and the molecular weight markers, in kilodaltons, are indicated on each immunoblot.

To consider whether H2B interacted directly with PARs, cell lysates from untreated or Plg-treated HUVEC lysates were subjected to pull-down experiments in which a GST-H2B construct was added to untreated or Plg-treated HUVEC lysates and the GST-pull-downs were analyzed by SDS-PAGE. Interaction of PAR1 (Figure 4A) and PAR2 (Figure 4B) was observed in the anti-GST eluates developed by immunoblotting with anti-PAR1 and anti-PAR2, respectively. While these interactions were evident for PAR1 and PAR2, we failed to detect an association of the H2B with β3 integrin (Figure 4C), a prominent transmembrane protein on HUVEC that can also function as a PlgR [23]. Thus, these data support an association of PAR1 and PAR2 with H2B, even though H2B lacks a transmembrane domain. In the experiments shown in Figure 4A,B, the association of H2B with the PARs was more pronounced in the Plg-treated HUVEC lysates compared to the cells not treated with Plg.

### 3.4. PARs Link H2B to Intracellular Signaling and Internalization Apparatus

**Figure 5:**
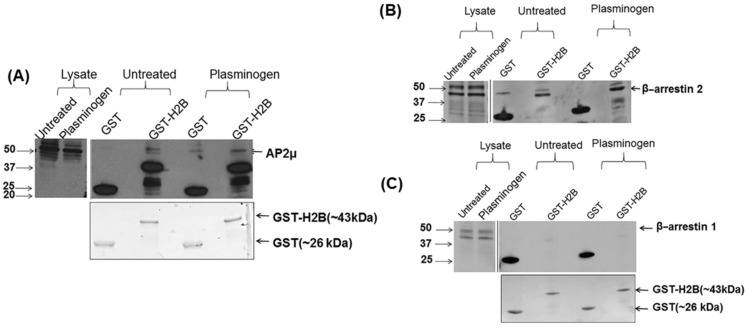
Association of H2B with clathrin adaptor AP2μ and β-arrestin 2 in HUVECs. In pulldown assays, GST-H2B substantially interacted with (**A**) clathrin adaptor AP2μ and (**B**) β-arrestin 2, in Plg-treated HUVECs compared to unstimulated HUVECs. (**C**) GST-H2B did not interact with β-arrestin 1 under these conditions. Loading of GST and GST-H2B is shown via Coomassie stained gels. “Lysate” denotes lysates of untreated or Plg-treated HUVECs. Mouse anti-AP2µ, mouse anti-β-arrestin 2 and mouse anti-β-arrestin 1 antibodies detected these proteins in immunoblots. Solid lines between gels denote the position of the molecular weight ladder and the molecular weight markers are indicated in kilodaltons.

PAR1 signaling and internalization involves the clathrin-adapter protein AP2. PAR2 does not interact with AP2 but rather with β-arrestin 2 for internalization [29,30]. As shown in Supplementary material Figure S1, PAR1 immunoprecipitated AP2μ in both untreated and Plg-treated HUVEC lysates, and this interaction was more pronounced in Plg-treated cell lysates [30]. PAR2 did not co-immunoprecipitate with AP2μ (Supplementary Materials Figure S1), which is consistent with its lack of the YXXΦ motif that is recognized by AP2μ [48]. ENO-1, another PlgR lacking a transmembrane domain, does not seem to interact with PAR1 or PAR2 under these conditions (Supplementary Materials Figure S2). Since H2B was found to associate with PAR1, which is internalized by AP2μ, we sought to investigate if H2B also associated with AP2μ. In GST-H2B pulldown assays, we found that H2B displayed a constitutive interaction with AP2μ, and Plg enhanced this interaction (Figure 5A). β-arrestins are recruited to the plasma membrane where they play an important role in the internalization of GPCRs by binding to clathrin and the clathrin adaptor AP2 complex [49], and they contribute to the desensitization and internalization of PAR2 [29,50]. Since H2B interacts with PAR2, we sought to determine if it also associates with β-arrestin. GST-H2B pulldown assays in HUVEC lysates showed that H2B associated with β-arrestin 2 both constitutively and upon Plg stimulation (Figure 5B). However, no such interaction was observed between H2B and β-arrestin 1 (Figure 5C) under similar conditions. This finding suggests a selective interaction of H2B with a β-arrestin 2-mediated signaling pathway.

## 4. Discussion

In a prior study [35], we demonstrated that H2B was present on HUVEC using its susceptibility to surface biotinylation as an approach. In the present study, this conclusion was supported by an independent approach, flow cytometry. Since apoptosis is regulated by Plg binding to receptors [47], our finding of H2B on the surface of single, viable endothelial cells, as established by live–dead staining, bolsters our conclusion that H2B can be an endothelial cell surface molecule. We further showed that H2B is expressed on endothelial cells of both venous and arterial origin (Figure 2). Based on flow cytometry, the extent of H2B surface expression on HUVEC appears to be low and similar to that of other PlgRs, ENO-1 and Annexin 2, by the same technique. However, the location of PlgRs relative to other cell-surface molecules may determine the ability of H2B and other PlgRs to make functional contributions.

Our data suggest that H2B, in its capacity as a PlgR, makes a significant contribution to an endothelial cell response. We first established that Plg and, more specifically, Plm generated from Plg exerts a major effect on endothelial cell migration. Under the conditions of the migration assay used, Plg enhanced migration by ~50%, and a specific plasmin inhibitor fully blunted this increase, which was indicative of specificity. We then showed that an antibody that blocks the binding of Plg to the region of H2B that harbors its C-terminal lysine, which engages Plg, reduced endothelial cell migration. In fact, the reduction in Plg-dependent HUVEC migration was diminished by 48.5%. Our previous study demonstrated the specificity of this antibody for the blockade of Plg binding to H2B and not to other PlgR including Enolase, Annexin 2 and p11. Each of these and still other PlgRs were previously shown to contribute to Plg-dependent responses in other cells, including tumor cells [7], but if and how these different PlgRs complement each other functionally remains to be resolved [35].

In our prior studies, we implicated PAR1 in responses that were dependent upon Plg binding to H2B in monocytoid cells [10,11]. In the present study, we found clear evidence of a role of the PARs in plasmin-dependent endothelial cell migration; antagonists of either PAR1 or PAR2 reduced this response. This effect was restricted to the migration that was enhanced by Plg, and the antagonists did not alter the Plg-independent migration of the cells. The involvement of PAR2 in H2B-/Plg-dependent endothelial cell migration was not expected. This is the first report where H2B was shown to utilize two GPCRs, PAR1 and PAR2, after stimulation by Plg. Of note, in endothelial cells, either a PAR1 or PAR2 antagonist completely inhibited the Plg-dependent migratory response. One potential explanation for the complete suppression of Plg-dependent endothelial cell migration by antagonists of either PAR may rest in their capacity to form and function as a heterodimer. The close proximity of PAR1 and PAR2 on the plasma membrane [42,51,52] may favor their heterodimerization or heterooligomerization [48,53], observed for PAR1 and PAR4 dimerization [54], upon Plm or thrombin activation of platelets [26,55]. Heteromerization may allow the Plm cleavage of PAR1, which can then transactivate PAR2 [40].

The association of H2B with PAR1 and PAR2 was demonstrated in pull down experiments. Plg enhanced this association (Figure 4A,B). Future studies will be undertaken to visualize the colocalization of these receptors on HUVECs and whether Plg treatment alters their distribution. Prospective studies will also aim to recapitulate these findings in vitro angiogenesis assays and in vivo animal models. In addition, H2B showed a similar trend in its association with AP2μ. The variability in degree of these associations may reflect the extent of plasminogen activation among experiments or differences in endothelial cells from different donors. AP2μ associates with the intracellular loops of PAR1, and β-arrestin 2 associates with those of PAR2, where they provide linkages of the GPCRs to the signaling and recycling machinery of the cells. Plasmin- and thrombin-induced activation leads to the internalization of PARs [51,52,56]. Whether H2B is also swept into the sensitization/resensitization cycles with the PARs remains to be investigated. Export to and retention on the cell surface, import into the nucleus and the associated functional consequences of H2B cycling have been reported [57,58]. In addition to internalization, H2B may be released from the cell surface and contribute to the extracellular pool of histones, which can also bind Plg and suppress local proteolysis [59].

## 5. Conclusions

Overall, our study is the first to link a transmembrane-lacking PlgR, H2B, to both PAR1 and PAR2 in endothelial cell function and to demonstrate that it may link H2B into the PAR internalization and signaling cascade. Histones have been implicated in a wide variety of diseases [60], such as hepatocellular carcinoma, where histones were found to promote the migration and invasion of cells [61]. Targeting H2B may represent a novel strategy to limit the contributions of the PARs to diseases including cancer where endothelial migration is important to disease progression.

## Data Availability

Not applicable.

## Supplementary Materials

The following are available online: https://www.mdpi.com/article/10.3390/biom12020211/s1, Figure S1: Association of PAR1 with clathrin adaptor AP2μ in HUVECs. Clathrin adaptor AP2μ associates with only PAR1, but not PAR2 in HUVECs in immunoprecipitation experiments. Mouse anti-AP2μ antibody detected Clathrin adaptor AP2μ in immunoblots. Solid line between gels denote where molecular weight ladder was run. Molecular weight markers in kilodaltons are indicated for every immunoblot. Figure S2: ENO-1 does not associate with PAR1 or PAR2. ENO-1, another PlgR lacking a transmembrane domain, does not engage PAR1 or PAR2 in HUVECs in immunoprecipitation experiments. Rabbit ENO-1 antibody detected ENO-1 in immunoblots. Solid line between gels denote where molecular weight ladder was run. Molecular weight markers in kilodaltons are indicated for every immunoblot.
