# Supplementary material for: Histone 2B Facilitates Plasminogen-Enhanced Endothelial Migration through Protease-Activated Receptor 1 (PAR1) and Protease-Activated Receptor 2 (PAR2)
Source: Biomolecules. 2022 Jan 26;12(2):211. doi: 10.3390/biom12020211 (PMC8961594; doi:10.3390/biom12020211)
Supplement: Supplementary file 1 [file biomolecules-12-00211-s001.zip › biomolecules-1537961-supplementary.pdf]

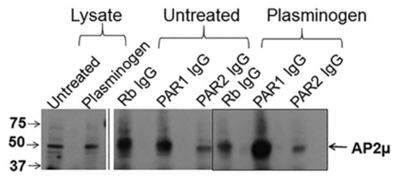

Figure S1. Association of PAR1 with clathrin AP2μ in HUVECs. Clathrin adaptor AP2μ associates with only PAR1, but not PAR2 in HUVECs in immunoprecipitation experiments. Mouse anti-AP2μ antibody detected Clathrin adaptor AP2μ in immunoblots. Solid line between gels denote where molecular weight ladder was run. Molecular weight markers in kilodaltons are indicated for every immunoblot.

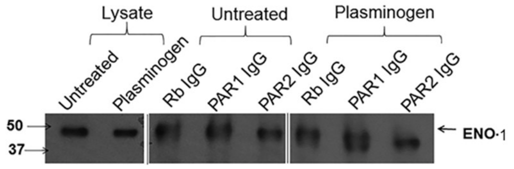

Figure S2. ENO-1 does not associate with PAR1 or PAR2. ENO-1, another PlgR lacking a transmembrane domain, does not engage PAR1 or PAR2 in HUVECs in immunoprecipitation experiments. Rabbit ENO-1 antibody detected ENO-1 in immunoblots. Solid line between gels denote where molecular weight ladder was run. Molecular weight markers in kilodaltons are indicated for every immunoblot. .
